# Lockdown due to COVID-19 in Spanish Children Up to 6 Years: Consequences on Diet, Lifestyle, Screen Viewing, and Sleep

**DOI:** 10.3389/ijph.2022.1604088

**Published:** 2022-06-03

**Authors:** Mercedes Díaz-Rodríguez, Jesús Carretero-Bravo, Celia Pérez-Muñoz, Mercedes Deudero-Sánchez

**Affiliations:** Department of Nursing and Physiotherapy, University of Cádiz, Cádiz, Spain

**Keywords:** obesity, Spain, COVID-19, childhood, lockdown, diet, lifestyle

## Abstract

**Objectives:** To record how the habits of children under 6 years of age in Cadiz have changed during lockdown, in order to identify those that could be a handicap for the problem of overweight and obesity.

**Methods:** We developed a new questionnaire to analyse family living habits. The questionnaire was administered online to parents of children aged zero-six years. Eating habits, sedentary lifestyles, screen viewing, and sleep changes were evaluated.

**Results:** Changes were found in family living habits, as shown by the results of McNemar’s tests (*p*-value < 0.01 in all pairs except one, *p*-value = 0.097). A worsening of habits was also found to be more accentuated in families with low income, low educational level or small size of home. Some positive aspects associated with family meals and parental involvement were found.

**Conclusions:** The lockdown has showed a significant break in the main routines of the children that could affect their health and may add to the worsening of the already poor childhood obesity situation. The positive aspects found can be instrumental in improving the situation in future similar situations.

## Introduction

SARS-CoV-2 (hereinafter COVID-19) emerged as a novel pathogen in late 2019 in China and quickly became a leading cause of morbidity and mortality worldwide [1]. Within months, the pandemic spread throughout the world, generating an unprecedented crisis. During this crisis, many countries have experienced a period of lockdown of the population, which has been forced to modify their lifestyle, being relegated to perform most tasks and activities within the home. In Spain, this situation lasted 98 days, after the establishment of the state of emergency decreed on 14th March 2020 [2], preventing citizens from continuing with their habits. The lockdown experienced in Spain was also one of the most restrictive in Europe, a fact that is reflected in some data such as electricity consumption [3].

Despite the new technologies, this situation of lockdown causes the population to perceive social isolation. Several studies have shown that social isolation worsens health problems [4] as well as alters cognitive function [5, 6] reducing the social signals that interact with us in choosing healthy habits can include eating, exercising, and sleeping habits [7–9].

Childhood obesity is a rapidly increasing health problem, with children being overweight or obese as early as age two [10]. The WHO recognizes it as a disease of epidemic character, which also has associated other pathologies [11]. This increased prevalence poses a problem for the health care system since a large proportion of children with obesity will become obese adults [12]. Therefore, the effects that lockdown may have on children’s habits will have a direct influence on their nutritional status.

Concerning these habits, their relationship with obesity is clear. The habitual consumption of snacks and sweetened drinks, of high caloric value, has been related to obesity already in the infantile age [13]. Also, the eating habits of the parents themselves influence those of their children, generating obesity where parental habits are not appropriate [14]. Regarding active living habits, there is strong evidence that increased screen viewing and sedentary habits are associated with overweight in young children and pre-schoolers [15].

Also, about physical activity, since childhood, there is a clear relationship with obesity. In young children and pre-schoolers, it has been proven that there is an inverse relationship between the time dedicated to active play and physical activity and measures of adiposity and risk of overweight [16].

Finally, irregular sleep patterns and fewer hours of sleep have been shown to be related to overweight and obesity in all age groups in childhood and adolescence [17].

Children up to the age of six are at a critical stage of development and any break in the routine that affects them can have significant effects on their health in the short, medium and long term [18]. Until now, most of the efforts directed to the prevention of overweight and obesity in children have been focused on those over 6 years old [19]. However, these efforts are not affecting on the rate of child obesity [20].

We wonder if the lockdown has been able to provoke changes in the routines and therefore in the habits of Spanish children, referring to food, physical activity, screen viewing and sleep, changes that could have direct consequences on their health. We have developed this research, whose objective is to record how the habits of children under 6 years of age in the province of Cadiz have changed during lockdown, in order to identify those that could be a handicap for the problem of overweight and obesity.

## Methods

### Design of the Studio and the Instrument

The following research question is formulated: How does lockdown due to COVID-19 affect the living habits of children under 6 years of age in the province of Cadiz?

This is an observational, descriptive, cross-sectional and prevalence study, which uses a questionnaire as a data collection tool.

The questionnaire is addressed to parents of children under 6 years of age. Given that the habits of life from birth to 6 years of age differ significantly, the questionnaire presents two different versions of the same. At the beginning of the questionnaire, an informed consent is also presented, in compliance with the data protection law [21].

For the development of the instrument, a previous review of the literature was carried out. The recommended values for the habits of interest were established, based on the standards of the World Health Organization [22], the National Sleep Foundation [23] and other recommendations from different Spanish organizations [24]. After this review, the instrument was developed by a team consisting of a doctor (a regular researcher on childhood obesity), a nurse and a mathematician. Due to the situation in which the questionnaire was constructed (during lockdown) no specific validation studies were carried out, although the questionnaire was previously passed to several parents who reviewed its content and provided their opinions.

The final design of the questionnaire consists of 55 questions, divided into sections as follows:

• Section one: made up of 14 questions that gather the sociodemographic characteristics.
• Sections two and three: consists of 18 common questions about eating and healthy living habits. At the same time, it has nine different questions for the two groups, four of them for children from zero-three years old and five for children of three-six years old.
• Section four contains 14 questions about changes in sleep habits.

The final construction includes 46 questions common to all parents and nine specifics to each age group.

### Data Collection

The questionnaire online format was chosen for data collection. It was designed using the Google Forms platform under the protection of an academic email account. For the dissemination of the questionnaire were used social networks, website of the research group, and the media of the province.

Inclusion criteria were parents and/or primary caregivers caring for children aged 6 years and under. To ensure that these criteria were met, they were clearly stated in the initial information and survey recipients were asked to respond to the survey only if they met them. The responses were then reviewed and those with any element of inconsistency were eliminated.

The questionnaire was distributed from 9th April 2020, until 1st May 2020, the dates on which the state of emergency was in effect in Spain and children under 14 were not allowed to leave their homes without a justified cause.

### Population and Sample Size

Initially, 789 responses were received. Of these, 535 corresponded to families indicating a zip code corresponding to the province of Cádiz, of which 478 were finally analysed after eliminating inconsistent responses and questionnaires that were not complete.

Reviewing the data from the latest census in the province of Cadiz, there are 122,600 children under 10 years, so if we extrapolate to children under 7 years is expected around 85,000 children [25]. With a Universe of 85,000 minors in a population that we assume to be heterogeneous, the sample that has been reached induces a margin of error of 4.5% in our findings.

### Study Variables

The variables of our study will be of two types: the sociodemographic or covariates and the questions of the questionnaire.

The sociodemographic variables of the study were divided as follows: discrete quantitative variables (number of children, persons in the home during lockdown), continuous variable (age of respondent) and qualitative variables (sex of the respondent, zip code, education level of the respondent, household income level, and square footage of the household during the lockdown).

The questions of the questionnaire are reflected in the Supplementary Appendix. As variables, they are qualitative with several options. The vast majority of them are written in such a way that the categories can be ordered from highest to lowest.

### Statistical Analysis

First, a descriptive analysis of the data obtained through the most frequent statistics was carried out. New groupings were made in those qualitative variables with categories lower than 5%.

For the analysis of the questions, those that in the opinion of the research team provided interesting results were reviewed. McNemar’s test was used for the analysis of differences between questions before and after lockdown.

In order to analyze the questions with the sociodemographic variables, the chi-square test was performed (using Fisher’s test if necessary). It was checked also if there is a linear association through Spearman’s linear correlation coefficient (ρ).

Eight questions of interest in habits after lockdown were grouped into two categories to visualize the results graphically and compared with the covariates also using the chi-square test and the Spearman correlation coefficient.

All the information obtained was transcribed to a computerized database, using the statistical program SPSS (version 24, licensed by the UCA), which allowed its subsequent statistical analysis taking as a level of significance the standard level 0.05.

### Ethics

The study was planned following the fundamental principles of the Helsinki Declaration [26] and following the primary Spanish data protection laws [21]. As an observational study, the recommendations made for the dissemination and presentation of results by the STROBE statement have been followed [27].

No ethics committee approval was required as the study does not involve any risk to the participants and no personal data is collected. The ethics committee of the province of Cadiz was consulted. Before starting to respond, it was mandatory to sign an informed consent form accepting participation.

## Results

### Description of the Sample

The summary of the sociodemographic variables can be seen in Table 1. Most women are observed, with 87.9% of the sample. Families with two children (48.7%) and parents whose youngest child is between three and 6 years old (60.9%) predominate. Almost half of those who responded had someone from the family unit who went out to work (49.8%).

**TABLE 1:** Study of sociodemographic characteristics of the sample (Cádiz, Spain. 2021).

Continuous variables		Media	SD
Age		37.01	5.60
**Discrete variables**		***N***	**(%)**
Sex	I prefer not to answer	3	0.6
	Woman	420	87.9
	Man	55	11.5
Number of children	One	200	41.8
	Two	233	48.7
	Three	36	7.5
	More than 3	9	1.9
People living at home	Two	10	2.1
	Three	171	35.8
	Four	217	45.4
	More than four	80	16.7
Going to work	Yes	238	49.8
	No	240	50.2
Level of studies	I prefer not to answer	8	1.7
	Primary	32	6.7
	Secondary	78	16.3
	Vocational training	139	29.1
	University students	221	46.2
Income level (in thousands of euros)	I prefer not to answer	101	21.1
	Less than 10	68	14.2
	Between 10 and 20	128	26.8
	Between 20 and 35	118	24.7
	Between 35 and 50	45	9.4
	More than 50	18	3.8
Size of the main residence (square meters)	I prefer not to answer	10	2.1
	Less than 75	96	20.1
	Between 75 and 100	232	48.5
	Between 100 and 150	101	21.1
	More than 150	39	8.2
Age of youngest child	Less than three	187	39.1
	Between three and six	291	60.9

Sociodemographic characteristics of the sample (*n* = 478).

Regarding the socioeconomic variables, 46.2% of those surveyed have university studies and 23% stay in primary and/or secondary studies. The majority of those surveyed earn between 10,000 and 20,000 € (26.8%). Finally, the majority group (48.5%) in the size of the main home is between 75 and 100 m^2^; only 8.2% of those surveyed have a home of more than 150 square meters.

### Questionnaire Questions Results

The three factors associated with obesity are considered important, although the most important is food (essential for 68.2%). The factor that is given the least importance (although high) is physical exercise and active lifestyle, with 53.8%.

The results of the remaining questions could be reviewed in Supplementary Appendix SA. Regarding the specific eating habits during lockdown, 56.3% of the respondents consider that these were similar to the previous ones, and 60.9% in the case of their children. An interest in improving the children’s nutrition at this stage was also observed (61.7%). With regard to impulsive eating associated with the pandemic, 45.2% of parents say that they have done this on a regular basis, with 29.7% of parents seeing this behaviour in their children.

Physical activity has been reduced, with 65.3% of respondents reporting less exercise. These respondents also claim to have frequently taken measures to prevent their children’s lack of exercise (57.7%), as 81.4% of them consider their children’s exercise to be less than usual. With regard to sleep, parents consider that both their habits and those of their children have changed (62.6% and 59.0% respectively). In addition, they showed a later bedtime in 70.3% of cases and considered their sleep to be of poorer quality in 53.4% of cases.

The results of the comparison of the habit questions before and after lockdown are shown in Table 2. Significant differences were obtained in responses to all of two questions that questioned pre-lockdown and lockdown behaviour, except for a pair of daytime sleep habits.

**TABLE 2:** McNemar’s pre-confined and post-confined question comparison test (Cádiz, Spain. 2021).

Pair of questions	Chi2	*p*-value	Pair of questions	Chi2	*p*-value
Q22–Q23 (eating snacks and fast food)	20.058	0.002*	Q51–Q53 (children physical exercise)	89.084	0.000*
Q28–Q29 (parental physical exercise)	49.858	0.000*	Q62–Q63 (parental nightly sleep)	59.117	0.000*
Q31–Q32 (encourage active lifestyle)	133.804	0.000*	Q64–Q65 (children nightly sleep)	34.448	0.000*
Q33–Q34 (daily hours of screen)	322.364	0.000*	Q66–Q67 (parental daytime sleep habits)	10.742	0.097
Q35–Q36 (using screens as entertainment	257.562	0.000*	Q68–Q69 (children daytime sleep habits)	60.545	0.000*

McNemar’s pre-confined and post-confined question comparison test.

### Associating Socio-Demographic Variables to Questions

Those questions that were considered to have interesting results were included in the analysis (shown in the Supplementary Appendix SA). A summary with the significantly findings of comparisons between the covariates and the questions can be seen in the Table 3 and the statistical analysis can be seen in Supplementary Appendix SB.

**TABLE 3:** Summary of findings for questions of interest (Cádiz, Spain. 2021).

Discrete variables	Habits	Findings
Number of children	Eating habits	Relationship to use of snacks before and after lockdown
	Screen viewing	Relationship to screen time in lockdown and use as entertainment before lockdown
	Physical exercise and active lifestyle	Relationship to promotion of physical exercise before lockdown
	Sleeping habits	—
People living at home	Eating habits	Findings very similar to those found in the number of children variable
	Screen viewing	
	Physical exercise and active lifestyle	
	Sleeping habits	
Going to work	Eating habits	Relationship to family meals after lockdown
	Screen viewing	—
	Physical exercise and active lifestyle	Relationship to exercise and children during lockdown
	Sleeping habits	Relationship with rest while sleeping after the emergency state
Sex	Eating habits	Relationship to family meals and impulse eating after lockdown
	Screen viewing	—
	Physical exercise and active lifestyle	Relationship to physical exercise for oneself and during lockdown and for children during lockdown
	Sleeping habits	Relationship with rest while sleeping after the emergency state
Age group	Eating habits	Relationship eating in family before lockdown and eating impulsively during
	Screen viewing	Screen time ratio after lockdown
	Physical exercise and active lifestyle	Relationship to physical exercise during lockdown
	Sleeping habits	Relationship to children’s bedtime after lockdown
Income level (in thousands of euros)	Eating habits	Relationship to impulse eating in lockdown and use of snacks before and after
	Screen viewing	Relationship to pre-containment screen viewing
	Physical exercise and active lifestyle	Relationship to physical exercise during lockdown
	Sleeping habits	—
Level of studies	Eating habits	Relationship to use of snacks and family meals before and during lockdown
	Screen viewing	Relationship to pre-containment screen use
	Physical exercise and active lifestyle	Relationship to physical exercise in lockdown and promotion of physical activity in children
	Sleeping habits	Relationship to children’s bedtime after lockdown
Size of the main residence (square meters)	Eating habits	Relationship to use of snacks before and during lockdown
	Screen viewing	Relationship to screen time before and after lockdown
	Physical exercise and active lifestyle	Relationship to exercise and children during lockdown
	Sleeping habits	Relationship with proper rest when sleeping

**FIGURE 1:**
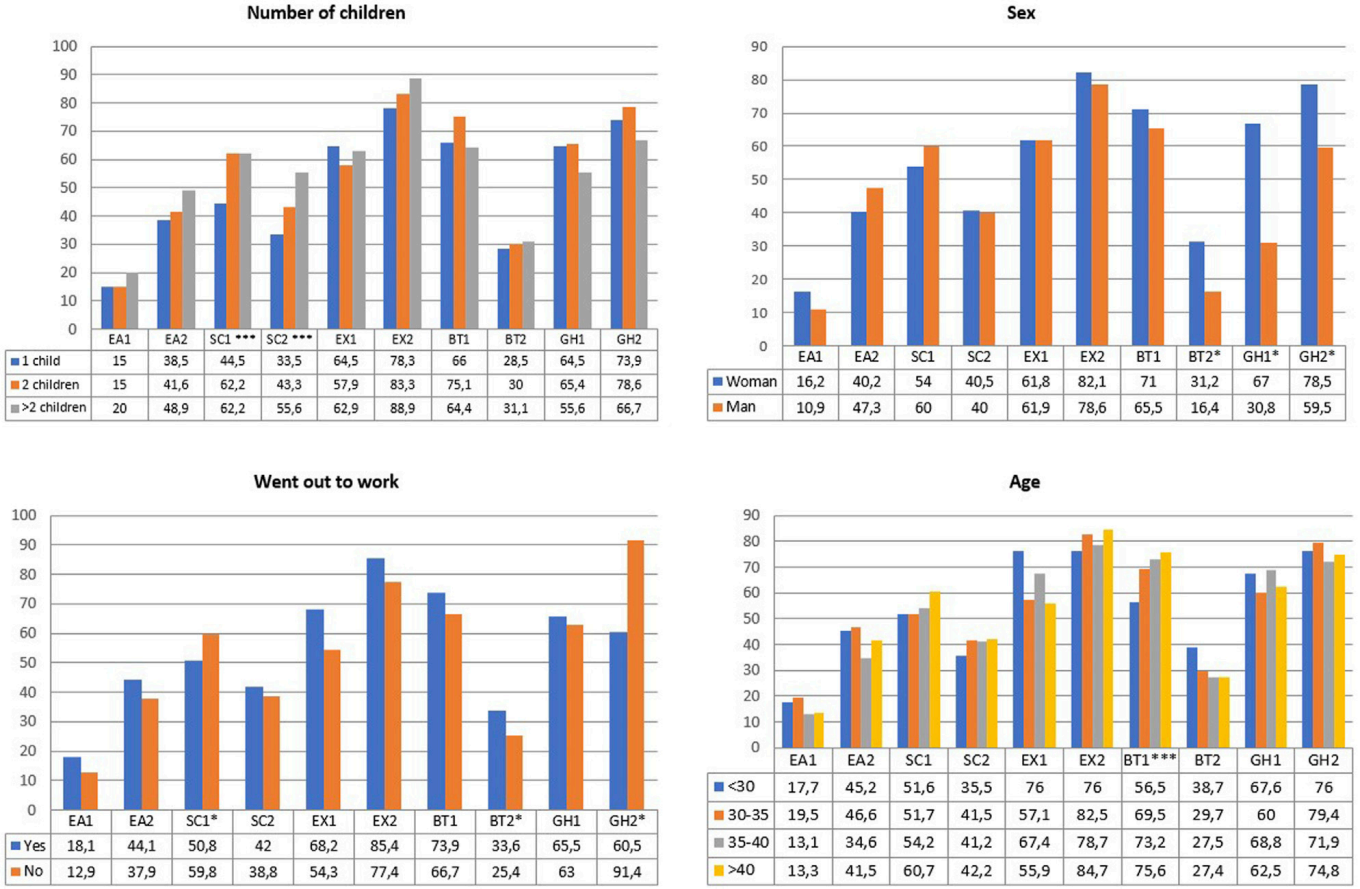
Risk factors in lockdown in relation to the number of children, sex of parents, parents went out to work, and age of the parents (Cádiz, Spain. 2021). Risk factors in lockdown in relation to the number of children, sex of parents, parents went out to work, and age of the parents. EA1: Feeding during lockdown worse, EA2: Avoid giving snacks infrequently, SC1: More than 2 h of screen per day, SC2: Use of screens for entertainment, EX1: Less than half an hour of exercise, EX2: Lower exercise during lockdown, BT1: Hour later at bedtime, BT2: Worst rest in sleep, GH1: To promote physical exercise, GH2: More family meals, *Significantly association, **Linear Association, ***Both Association.

**FIGURE 2:**
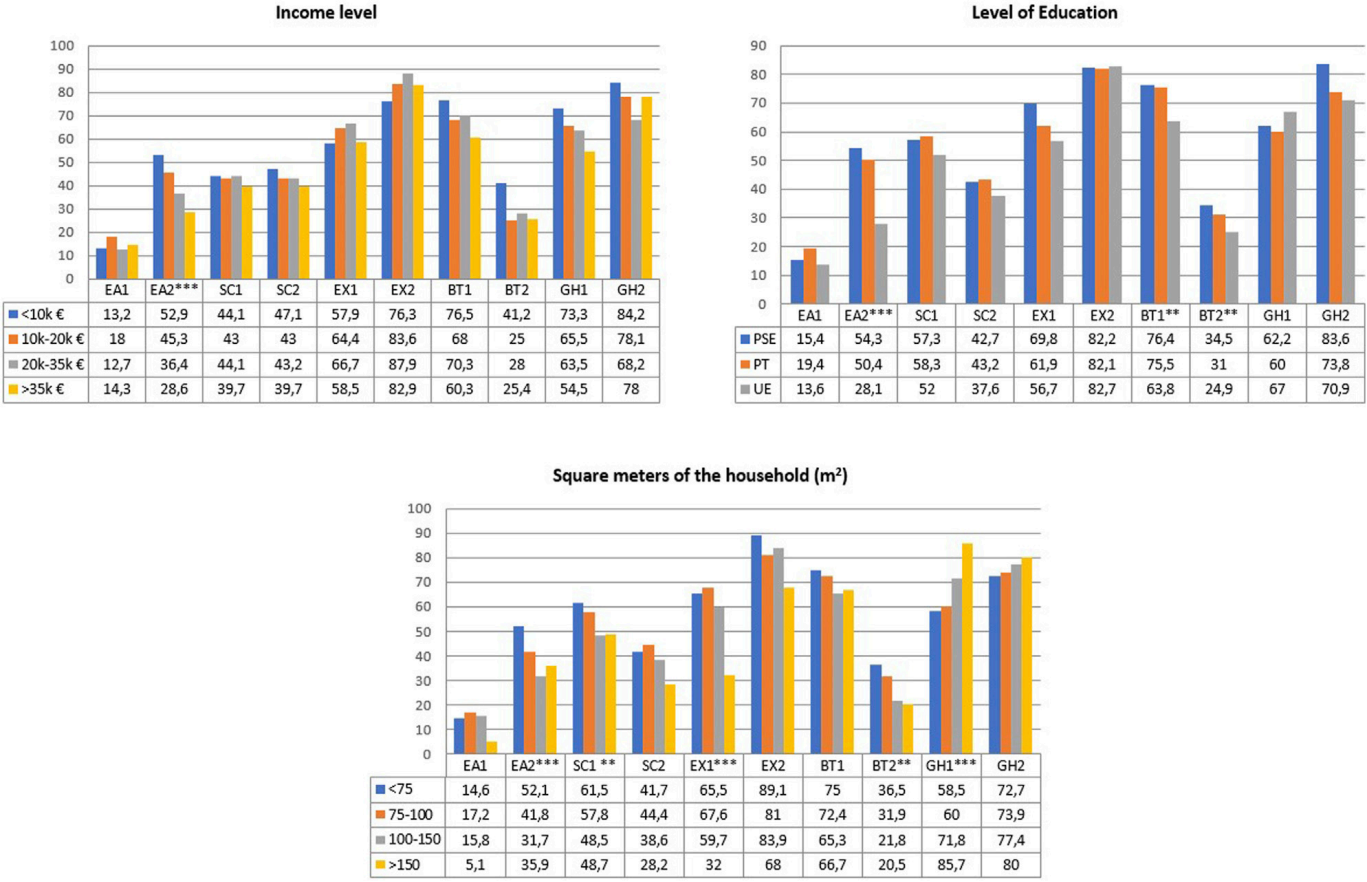
Risk factors in lockdown in relation to the income level, parents’ level of education and square meters of the household (Cádiz, Spain. 2021). Risk factors in lockdown in relation to the number of children, sex of parents, parents went out to work, and age of the parents. EA1: Feeding during lockdown worse, EA2: Avoid giving snacks infrequently, SC1: More than 2 h of screen per day, SC2: Use of screens for entertainment, EX1: Less than half an hour of exercise, EX2: Lower exercise during lockdown, BT1: Hour later at bedtime, BT2: Worst rest in sleep, GH1: To promote physical exercise, GH2: More family meals, PSE: Primary and Secondary Education, PT: Professional Training, UE: University Studies *Significantly association, **Linear Association, ***Both Association.

Additionally, groupings were made in eight questions in habits after lockdown, grouping them into two response categories and comparing them with the covariates. The figures associated with the comparisons are shown in Figures 1,2. In this last comparison and the statistical analysis in Supplementary Appendix SC, the variable number of people at home was discarded because of its high correlation with the number of children (*r* = 0.728, *p*-value < 0.001).

The number of children in the family unit influences significantly in screen viewing. The greater the number of children, the greater the screen viewing (more than 2 h with one child 44.5%, with more than two children 62.2%, *p*-value = 0.001). There are also influences on the use of screens as entertainment in lockdown: with one child 33.5%, but with more than two children 55.6% (*p*-value = 0.011).

The sex of the parents is somewhat more influential before lockdown but as a result of lockdown, mothers observe a worse sleep rest for their children than fathers (16.4% in man, 31.2% in woman, *p*-value = 0.023). Mothers also show a greater improvement in two good habits after lockdown, promotion of physical activity (66.7% in women, 30.8% in men, *p*-value = 0.009) and family meals as a family (78.5% in women, 59.5% in men, *p*-value = 0.008).

Parents who worked from home or were unemployed were able to participate more in family meals (91.4% compared to 60.5% of those who worked, *p*-value < 0.001), and 68.2% of parents who worked away from home reported that their children did less than half an hour of exercise, compared to 54.3% of those who worked from home (*p*-value = 0.015). Also, the parents who were at home had a superior use of screens in their children (more than 2 h, 59.8% in parents who were at home, 50.8% otherwise, *p*-value = 0.045).

With respect to the age of the parents, older parents allowed their children to go to bed later during lockdown, with the group over 40 years old stating that 75.6% of their children went to bed later, compared to 56.5% in the group of younger parents (*p*-value = 0.039).

The income level of parents has a particular influence on aspects associated with eating habits and physical activity. Low-income level has more difficulty avoiding the use of snacks for their children (52.9% of parents with lower income did not avoid the use of snacks, this percentage being 28.6% in the case of parents with higher income, *p*-value = 0.018).

Related to academic level of parents, university-educated parents are by far the most likely to avoid snacking in their children (28.1% versus 52.3% in case of non-university education, *p*-value < 0.001). Concerning sleep, the bedtimes of children of university parents have been 63.8% later, compared to non-university educated parents with 76% (*p*-value = 0.016).

Finally, the square meters of the family’s living space have positive influences on many of the changes in lockdown. The more square metres, the less snacking (up to 20.4% less in houses between 100 and 150 m^2^, *p*-value = 0.020), less screen overuse (48.7% vs. 61.5% in smaller houses, *p*-value = 0.008), more physical activity in children and more promotion of such activity (up to 27.2% higher in houses over 150 m^2^, *p*-value = 0.043). In addition, children’s sleep was 20.5% worse in the case of houses larger than 150 m^2^, compared to 36.5% in houses smaller than 75 m^2^ (*p*-value = 0.009).

## Discussion

### Global Analysis of Results

Lockdown has caused drastic changes in family living habits, as shown by the results of McNemar’s tests. Studies have already shown an increase in obesity in children aged 2–11 years associated with the pandemic [28]. Several aspects of changes are noteworthy and are discussed below.

After lockdown, parents report a considerable increase in screen use in their children. Other studies in our country also show a big increase [29, 30]. This exposure time exceeds the limits set by the WHO [22]. In addition to these studies, there is an increase in the use of screens for entertainment in 35.8% of cases.

Regarding physical exercise, parents show a considerable reduction in the hours of activity, with half an hour or less being the most frequent category far from the recommendations [22]. There is also a considerable reduction in physical exercise in children aged three to 6 years. This fact has two clear components: the risk of less physical exercise in children and the risk of observing in their parent’s habits of less physical exercise. Another study in Spain shows similar results to ours [30]. This fact seems to be generalized in all places with lockdown since in a study carried out in Italian children with obesity it is shown as the sport carried out lowers in lockdown [31].

The use of unhealthy snacks and meals in children increased after lockdown. This percentage of change is similar to that proposed by other studies [29]. Lockdown has also caused parents to eat impulsively sometimes which can become a problem since children will be able to observe inappropriate behaviors.

However, feeding presents some positive data in our study. There has been a significantly increase in the participation in family meals after lockdown. Studies suggest that families who regularly eat together achieve better nutritional health for their children [32]. This fact may offset the other negative factors associated with the diet found.

We found that before the lockdown the children slept fewer hours of sleep than recommended [23] while increased slightly during the lockdown, although without fulfilling the recommendations. It is also verified that the quality of dream of the parents is worse during the lockdown. References from other studies and sleep in lockdown show similar results [30, 31].

### Analysis of Sociodemographic Covariates

The number of children in the family unit influences screen viewing, eating habits and physical exercise. The greater the number of children, the greater the screen viewing and the worse the consumption of snacks and unhealthy foods. There are also on the use of screens as entertainment in lockdown.

In another study it is observed that eating habits are better in families with more than seven members [33] although the study associates this improvement to a greater extent with the lack of accessibility to fast food. These findings invite us to propose protection policies aimed at reconciling families, allowing large families in possible future situations like this.

The sex of the parents is somewhat more influential before lockdown, although women show even more significant improvement in two aspects of beneficial lockdown, promotion of physical activity and family meals. As a result of lockdown, mothers observe a worse sleep rest for their children than fathers.

The fact that mothers show an improvement in these beneficial aspects may be related to a greater workload in household and childcare tasks than fathers, as shown in another Spanish study [34].

Our study also looked if respondents had to leave for work during lockdown. Parents who stay at home were able to participate more in family meals, as well as improve their children’s physical exercise. In addition, parents who worked telematically considered their children’s sleep rest to be less bad than those parents who worked.

Given these results, it is essential analyze how the established teleworking can positively influence certain parental practices. As pointed out by UNICEF in its recommendations [35], it is necessary to establish measures that allow the reconciliation of family and work in the situation arising from the pandemic. Studies show the extent of teleworking during lockdown as an opportunity to reduce costs and to generate a favourable climate of reconciliation [36]. However, its regulation in family life must be adequate, since some studies on lockdown show how Spanish mothers [34] who telework continue to spend more time on the main household tasks than men, even though they spend similar times at work.

The income level of parents has a particular influence on aspects associated with eating habits and physical activity, but the latter only before lockdown. Low-income level has more difficulty avoiding the use of snacks for their children, in addition to eating more impulsively than parents with a higher income level.

This fact is critical in the known correlation between poverty and obesity [37]. Parents with fewer resources resort to this type of high-calorie foods, in addition to showing their children less appropriate eating habits.

Parents’ level of education is a variable that shows a high level of association with various questions. Time of physical activity of children during lockdown did show significant influences in our study on the academic level of parents. This influence can also be seen to occur both before and after lockdown has an even more significant influence [38].

The academic level of parents has also influenced eating habits. College-educated parents are by far the most likely to avoid snacking in their children. The OPIK study [29] mentioned above also shows that the lower educational level of parents is related to lower intake of fruits and vegetables. Another study [33] shows that the mother’s educational level is positively correlated with the consumption of fruits and vegetables and negatively with the consumption of sugary drinks and fast food.

Concerning sleep, bedtime has been earlier in parents with higher educational level. This data shows that the level of training of the parents can influence positively in the sleep habits of their children, as other studies show [39].

Finally, the square meters of the family’s main home have a clear influence: the more square meters, the more exercise is done in lockdown, both by the parents and by the children. It is also observed that sleep rest is somewhat better in those respondents who say they have a larger home. In feeding, the influence is similar to that of the level of studies.

This improvement in physical activity may in turn be associated with lower screen viewing compared to families with a smaller home. In turn, children in these families have an improvement in nutrition, which also occurs by educational level and income level. The OPIK group adds to our findings data such as that 25.9% of the child population in Spain have no outdoor spaces in their homes. In addition, low-income families have had during lockdown greater lack of natural light, humidity problems in their homes, and noise problems [29].

In view of the evidence of these data, awareness and social protection campaigns are once again necessary to ensure that the recommendations on physical activity, diet, screen viewing and sleep are appropriate to avoid obesity, reaching all strata of society, especially families with a lower level of education, low income, and small homes with no open spaces.

### Limitations

One of the main advantages of our study was that we took a sample of parents from the province of Cadiz with adequate size, with a relatively small margin of error in that population (4.5%). However, its results can only be extrapolated to that province.

On the other hand, given the age of the children of interest in the study, it was the parents who answered the questions. This may lead to bias in the information parents may provide, as suggested by other studies [40]. Another bias may be the use of the online questionnaire, which is necessary in the confinement situation, but limits access to families with internet at home.

Finally, as it is an observational study, the conclusion that can be derived from it do not show causality in any case, although in the situation derived from the pandemic it was complicated to build a similar study that could show some causal result.

### Conclusion

In conclusion, our study has shown the lockdown has showed a significant break in the main habits and routines that could affect children health in the short and medium-term.

In a context in which the problem of obesity is already quite worrying, findings have been mostly negative, affecting factors such as worse eating habits, less physical activity in children or increased viewing of screens. However, some positive aspects derived from lockdown have been detected, such as an increase in family meals or the influence of teleworking in trying to mitigate the effects of lockdown. This invites us to see the situation of lockdown as an excellent opportunity to improve the interaction between parents and children.

Since our study has detected an even more pronounced worsening of habits in families with a low social level, social protection must play a crucial role in addressing the vulnerabilities of families to poverty, inequality and deprivation. We have to influence in greater prevention facing future situations of lockdown, avoiding the rupture of behaviour habits and promoting healthy lifestyles associated with the WHO recommendations.
