# Supplementary material for: Lockdown due to COVID-19 in Spanish Children Up to 6 Years: Consequences on Diet, Lifestyle, Screen Viewing, and Sleep
Source: Int J Public Health. 2022 Jun 3;67:1604088. doi: 10.3389/ijph.2022.1604088 (PMC9203684; doi:10.3389/ijph.2022.1604088)
Supplement: Supplementary file 1 [file DataSheet1.docx]

Appendices – Appendix A: Results of the habits in lockdown questionnaire (Cádiz, Spain. 2021)

Table 1 - Common questions, eating habits and active lifestyle sections (Cádiz, Spain. 2021)

Table 2 - Sleeping Habits Section Questions (Cádiz, Spain. 2021)

Table 3 - Specific questions, eating habits and active lifestyle sections (Cádiz, Spain. 2021)

Appendix B: Chi-square test results (Cádiz, Spain. 2021)

Table 1: Summary of findings for questions of interest (Cádiz, Spain. 2021)

Appendix C: Additional data (Cádiz, Spain. 2021)

Figure 1 – Risk factors in lockdown in relation to the number of children (Cádiz, Spain. 2021)

Figure 2 – Risk factors in lockdown in relation to the sex of the parents (Cádiz, Spain. 2021)

Figure 3 – Risk factors in lockdown in relation to whether the respondent went out to work (Cádiz, Spain. 2021)

Figure 4 – Risk factors in lockdown in relation to the age of the parents (Cádiz, Spain. 2021)

Figure 5 – Risk factors in lockdown in relation to income level (Cádiz, Spain. 2021)

Figure 6 – Risk factors in lockdown in relation to the level of education (Cádiz, Spain. 2021)

Figure 7 – Risk factors in lockdown in relation to the square meters of the main residence (Cádiz, Spain. 2021)

**Appendix A: results of the habits in lockdown questionnaire**

**Table 1:** Common questions, eating habits and active lifestyle sections

| Q16/Q38 How important do you think healthy eating is in the development of your children (compared to other factors such as study, physical activity, social environment, sleep...)? (1) | Same as the other factors | 11.9% | Q27/Q49 How important do you consider physical exercise and an active lifestyle to be in the development of your children (compared to other factors such as study, diet, social environment, sleep...)? (1) | Less important | 0.4% |
| --- | --- | --- | --- | --- | --- |
|  | Important | 19.9% |  | Same as the other factors | 14.6% |
|  | Very important | 68.2% |  | Important | 31.2% |
| Q18/Q41 Considers that during lockdown their eating habits with respect to the usual ones are | Worse | 1.5% |  | Very important | 53.8% |
|  | Slightly worse | 18.8% | Q28/Q50 Prior to lockdown, did you engage in any physical exercise? (2) | I didn't exercise | 32.2% |
|  | Same | 56.3% |  | Yes, less than half an hour a day | 12.6% |
|  | Something better | 14.0% |  | Yes, between half an hour and an hour a day | 45.0% |
|  | Best | 9.4% |  | Yes, more than one hour a day | 10.3% |
| Q19/Q42 Considers that during the lockdown the feeding of their children with respect to the usual one is being | Worse | 0.6% | Q29/Q52 Are you doing any physical exercise in your home during lockdown? (2) | I am not exercising | 37.4% |
|  | Something worse | 14.9% |  | Yes, less than half an hour a day | 24.3% |
|  | Same | 60.9% |  | Yes, between half an hour and an hour a day | 34.1% |
|  | Something better | 12.6% |  | Yes, more than one hour a day | 4.2% |
|  | Best | 11.1% | Q30/Q54 During lockdown, you consider that your physical exercise is being: (2) | Less than what you usually do | 65.3% |
| Q20/Q43 Are you trying to take steps during lockdown to improve your children's nutrition | No | 16.9% |  | Same as the one you usually do | 17.6% |
|  | I have not considered it | 21.3% |  | Superior to what you usually do | 17.2% |
|  | Yes | 61.7% | Q33/Q56 Before lockdown, your children's daily hours of screen viewing (cell phones, tablets, television...) used to be: (2) | Less than 1 hour | 46.7% |
| Q21/Q44 Have you had any problems during your lockdown in providing essential food for your children? | Sometime | 20.9% |  | Between 1 and 2 hours | 47.7% |
|  | On several occasions | 6.1% |  | More than 2 hours | 5.6% |
|  | No | 73.0% | Q34/Q57 During lockdown, your children's daily hours of screen viewing (cell phones, tablets, television...) are usually: (2) | Less than 1 hour | 11.1% |
| Q22/Q45 Prior to lockdown, you prevented your children from eating snacks, fast food, and high-calorie foods: (2) | Never | 2.9% |  | Between 1 and 2 hours | 34.1% |
|  | Rarely | 4.6% |  | More than 2 hours | 54.8% |
|  | Sometimes | 25.7% | Q35/Q58 Before the lockdown, I used the screens (cell phones, tablets, television...) to entertain your children (2) | Never | 19.2% |
|  | Frequently | 46.2% |  | Hardly ever | 5.9% |
|  | Always | 20.5% |  | On a few occasions | 39.5% |
| Q23/Q46 During lockdown, prevent your children from eating snacks, fast food, and high-calorie foods: (2) | Never | 2.5% |  | Sometimes | 30.8% |
|  | Rarely | 7.1% |  | Frequently | 4.6% |
|  | Sometimes | 31.4% | Q36/Q59 During lockdown, you use the screens (cell phones, tablets, television...) to entertain your children (2) | Never | 1.7% |
|  | Frequently | 41.4% |  | Hardly ever | 6.7% |
|  | Always | 17.6% |  | On a few occasions | 11.3% |
| Q24/Q47 Considers that the situation resulting from the lockdown makes him eat impulsively: (2) | Never | 18.8% |  | Sometimes | 40.0% |
|  | Sometime | 36.0% |  | Frequently | 40.4% |
|  | Sometimes | 32.4% | Q37/Q60 During lockdown, you are taking measures to avoid possible inactivity of your children (games, exercises...) | Never | 0.4% |
|  | Frequently | 12.8% |  | Rarely | 5.4% |
| Q25/Q48 Considers that the situation resulting from the lockdown makes their children eat in an impulsive way: | Never | 40.0% |  | Sometimes | 36.4% |
|  | Sometime | 30.3% |  | Frequently | 57.7% |
|  | Sometimes | 20.3% | 1: Questions about the importance of habits  2: Questions with results of interest according to the research team | | |
|  | Frequently | 9.4% |  |  |  |

**Table 2**: Sleeping Habits Section Questions

| Q61 How important do you consider sleep habits and routines to be in the development of your children (in comparison with other factors such as study, diet, social environment, physical activity... )? (1) | Very unimportant | 0.2% | Q68 Before the alarm state, your children slept during the day for the next few hours: | They did not sleep during the day | 57.9% |
| --- | --- | --- | --- | --- | --- |
|  | Less important | 0.4% |  | Less than half an hour | 3.6% |
|  | Same as the other factors | 11.7% |  | Between half an hour and one hour | 15.1% |
|  | Important | 23.6% |  | Between one hour and two hours | 18.6% |
|  | Very important | 64.0% |  | More than two hours | 4.8% |
| Q62 Before the alarm state, your nightly sleep hours were: | Less than 6 hours | 6.9% | Q69 During the alarm state, your children sleep during the day for the next few hours: | They do not sleep during the day | 69.0% |
|  | Between 6 and 7 hours | 36.2% |  | Less than half an hour | 5.0% |
|  | Between 7 and 8 hours | 41.6% |  | Between half an hour and one hour | 8.4% |
|  | More than 8 hours | 15.3% |  | Between one hour and two hours | 13.4% |
| Q63 During the alarm state, your nightly sleeping hours are: | Less than 6 hours | 17.6% |  | More than two hours | 4.2% |
|  | Between 6 and 7 hours | 26.2% | Q70 Do you think that your sleep routines have changed since lockdown? | Yes | 62.6% |
|  | Between 7 and 8 hours | 35.4% |  | No | 37.4% |
|  | More than 8 hours | 20.9% | Q71 Do you think your children's sleep routines have changed since lockdown? | Yes | 59.0% |
| Q64 Before the alarm state, your children's nightly sleeping hours were: | Less than 6 hours | 1.5% |  | No | 41.0% |
|  | Between 6 and 7 hours | 3.3% | Q72 After the alarm condition, the time your youngest child goes to bed (2) | It is before the usual | 1.9% |
|  | Between 7 and 8 hours | 11.7% |  | It's the same time | 27.8% |
|  | Between 8 and 9 hours | 39.1% |  | It is after the usual | 70.3% |
|  | More than 9 hours | 44.4% | Q73 Considers that after the state of alarm, his rest while sleeping is: (2) | Something worse | 19.5% |
| Q65 During the alarm state, your children's nightly sleeping hours are: | Less than 6 hours | 3.3% |  | Worse | 33.9% |
|  | Between 6 and 7 hours | 7.9% |  | Same | 34.5% |
|  | Between 7 and 8 hours | 11.5% |  | Something better | 6.9% |
|  | Between 8 and 9 hours | 29.5% |  | Best | 5.2% |
|  | More than 9 hours | 47.7% | Q74 Considers that, after the state of alarm, the rest of your children while sleep is: | Something worse | 8.8% |
| Q66 Before the alarm state, I slept during the day for the next few hours: | Did not sleep during the day | 75.5% |  | Worse | 20.7% |
|  | Less than half an hour | 9.2% |  | Same | 58.8% |
|  | Between half an hour and one hour | 11.5% |  | Something better | 6.7% |
|  | More than one hour | 3.8% |  | Best | 5.0% |
| Q67 During the alarm state, sleeps during the day for the next few hours: | Does not sleep during the day | 74.7% | 1: Questions about the importance of habits  2: Questions with results of interest according to the research team | | |
|  | Less than half an hour | 9.6% |  |  |  |
|  | Between half an hour and one hour | 13.2% |  |  |  |
|  | More than one hour | 2.5% |  |  |  |

**Table 3:** Specific questions, eating habits and active lifestyle sections

| CHILDREN BETWEEN 3 and 6 YEARS (n=291) | | | CHILDREN UNDER 3 YEARS OF AGE (n=187) | | |
| --- | --- | --- | --- | --- | --- |
| Q39 Before lockdown, the family unit used to eat together: (3) | No daily meals | 3.1% | Q17 In your opinion, having the family unit perform the daily meals at the same time and in the same location is | Unimportant | 0% |
|  | At some daily meal | 62.9% |  | Something important | 4.8% |
|  | At all daily meals | 34.0% |  | Important | 38.0% |
| Q40 During lockdown, the family unit usually eats together (3) | No daily meals | 0.7% |  | Very important | 57.2% |
|  | At some daily meal | 24.0% | Q26 If any of your children continue to breastfeed or formula feed and this is their primary means of feeding, do you feel that their breastfeeding habits have changed during lockdown? | I don't have a nursing child | 31.0% |
|  | At all daily meals | 75.3% |  | No, they stay the same | 50.8% |
| Q51 Prior to lockdown, did your children engage in any physical exercise outside of school hours? (2) | They did not exercise | 12.7% |  | Yes, they have changed for the worse | 8.0% |
|  | Yes, less than half an hour a day | 13.5% |  | Yes, they have changed for the better | 10.2% |
|  | Yes, between half an hour and an hour a day | 51.5% | Q31 Before lockdown, did you try to encourage your children to have an active lifestyle with games and activities at home? (3) | On a few occasions | 3.2% |
|  | Yes, more than one hour a day | 22.3% |  | Sometimes | 11.8% |
| Q53 Are your children doing any physical exercise at home during lockdown? (2) | They are not exercising | 25.1% |  | Frequently | 26.7% |
|  | Yes, less than half an hour a day | 36.4% |  | Whenever possible | 58.3% |
|  | Yes, between half an hour and an hour a day | 30.6% | Q32 During lockdown, do you try to encourage your children to have a active living with games and activities at home? (3) | On a few occasions | 0% |
|  | Yes, more than one hour a day | 7.9% |  | Sometimes | 6.9% |
| Q55 During lockdown, do you consider that the physical exercise your children are doing is: (2) | Less than what they usually do | 81.4% |  | Frequently | 28.9% |
|  | Same as the one they usually perform | 13.4% |  | Whenever I can | 64.2% |
|  | Superior to what they usually do | 5.2% | 1: Questions about the importance of habits  2: Questions with results of interest according to the research team  3: Questions with positive results of interest according to the research team | | |

**Appendix B: chi-square test results**

| **Table 1:** Summary of findings for questions of interest | | | |
| --- | --- | --- | --- |
| Discrete variables | | Habits | *Findings* |
| Number of children | Eating Habits | | *Relationship to use of snacks before and after confinement* |
|  | Screen Viewing | | *Relationship to screen time in confinement and use as entertainment before confinement* |
|  | Physical Exercise and Active Lifestyle | | *Relationship to promotion of physical exercise before confinement* |
|  | Sleeping Habits | | *-* |
| People living at home | Eating Habits | | *Findings very similar to those found in the number of children variable* |
|  | Screen Viewing | |  |
|  | Physical Exercise and Active Lifestyle | |  |
|  | Sleeping Habits | |  |
| Going to work | Eating Habits | | *Relationship to family meals after confinement* |
|  | Screen Viewing | | *-* |
|  | Physical Exercise and Active Lifestyle | | *Relationship to exercise and children during confinement* |
|  | Sleeping Habits | | *Relationship with rest while sleeping after the alarm state* |
| Sex | Eating Habits | | *Relationship to family meals and impulse eating after confinement* |
|  | Screen Viewing | | *-* |
|  | Physical Exercise and Active Lifestyle | | *Relationship to physical exercise for oneself and during confinement and for children during lockdown* |
|  | Sleeping Habits | | *Relationship with rest while sleeping after the alarm state* |
| Age group | Eating Habits | | *Relationship eating in family before confinement and eating impulsively during* |
|  | Screen Viewing | | *Screen time ratio after confinement* |
|  | Physical Exercise and Active Lifestyle | | *Relationship to physical exercise during confinement* |
|  | Sleeping Habits | | *Relationship to children's bedtime after confinement* |
| Income level (in thousands of euros) | Eating Habits | | *Relationship to impulse eating in confinement and use of snacks before and after* |
|  | Screen Viewing | | *Relationship to pre-containment screen viewing* |
|  | Physical Exercise and Active Lifestyle | | *Relationship to physical exercise during confinement* |
|  | Sleeping Habits | | *-* |
| Level of studies | Eating Habits | | *Relationship to use of snacks and family meals before and during confinement* |
|  | Screen Viewing | | *Relationship to pre-containment screen use* |
|  | Physical Exercise and Active Lifestyle | | *Relationship to physical exercise in confinement and promotion of physical activity in children* |
|  | Sleeping Habits | | *Relationship to children's bedtime after confinement* |
| Size of the main residence (square meters) | Eating Habits | | *Relationship to use of snacks before and during confinement* |
|  | Screen Viewing | | *Relationship to screen time before and after confinement* |
|  | Physical Exercise and Active Lifestyle | | *Relationship to exercise and children during confinement* |
|  | Sleeping Habits | | *Relationship with proper rest when sleeping* |

**Appendix C:** Aditional data

**Figure 1.**

† Risk factors in lockdown in relation to the number of children.

**Figure 2.**

† Risk factors in lockdown in relation to the sex of the parents.

**Figure 3.**

† Risk factors in lockdown in relation to whether the respondent went out to work.

**Figure 4.**

† Risk factors in lockdown in relation to the age of the parents.

Figure 5.

† Risk factors in lockdown in relation to income level.

**Figure 6.**

† Risk factors in lockdown in relation to the level of education.

Figure 7.

†Risk factors in lockdown in relation to the square meters of the main residence.
